# Levosimendan in acute heart failure with severely reduced kidney function, a propensity score matched registry study

**DOI:** 10.3389/fcvm.2022.1027727

**Published:** 2022-10-20

**Authors:** Felix Arne Rottmann, Ann Katrin Breiden, Xavier Bemtgen, Thomas Welte, Alexander Supady, Tobias Wengenmayer, Dawid Leander Staudacher

**Affiliations:** ^1^Interdisciplinary Medical Intensive Care, Medical Center, Faculty of Medicine, University of Freiburg, Freiburg, Germany; ^2^Department of Medicine IV – Nephrology and Primary Care, Faculty of Medicine and Medical Center, University of Freiburg, Freiburg, Germany; ^3^Friedrich Miescher Institute for Biomedical Research, Basel, Switzerland; ^4^Heidelberg Institute of Global Health, University of Heidelberg, Heidelberg, Germany

**Keywords:** acute heart failure, kidney failure, reduced kidney function, levosimendan, ICU, survival

## Abstract

**Background:**

Patients with heart failure frequently present with kidney dysfunction. Kidney function is relevant, as prognosis declines with reduced kidney function and potentially beneficial drugs like levosimendan are contraindicated for missing safety data.

**Materials and methods:**

A single-center retrospective registry study was conducted including all patients receiving levosimendan on a medical intensive care unit between January 2010 and December 2019. Exclusion criteria were a follow-up less than 24 h or missing glomerular filtration rate (eGFR) before administration of levosimendan. The first course of treatment was evaluated. Patients were stratified by eGFR before drug administration and the primary endpoint was a composite of supraventricular-, ventricular tachycardia and death within 7 days after administration of levosimendan. An internal control group was created by propensity score matching.

**Results:**

A total of 794 patients receiving levosimendan were screened and 368 unique patients were included. Patients were predominantly male (73.6%) and median age was 63 years. Patients were divided by eGFR into three groups: >60 ml/min/1.73 m^2^ (*n* = 110), 60–30 ml/min/1.73 m^2^ (*n* = 130), and <30 ml/min/1.73 m^2^ (*n* = 128). ICU survival was significantly lower in patients with lower eGFR (69.1, 57.7, and 50.8%, respectively, *p* = 0.016) and patients with lower eGFR were significantly older and had significantly more comorbidities. The primary combined endpoint was reached in 61.8, 63.1, and 69.5% of subjects, respectively (*p* = 0.396). A multivariate logistic regression model suggested only age (*p* < 0.020), extracorporeal membrane oxygenation (*p* < 0.001) or renal replacement therapy (*p* = 0.028) during day 1–7 independently predict the primary endpoint while kidney function did not (*p* = 0.835). A propensity score matching of patients with eGFR < 30 and >30 ml/min/1.73 m^2^ based on these predictors of outcome confirmed the primary endpoint (*p* = 0.886).

**Conclusion:**

The combined endpoint of supraventricular-, ventricular tachycardia and death within 7 days was reached at a similar rate in patients independently of kidney function. Prospective randomized trials are warranted to clarify if levosimendan can be used safely in severely reduced kidney function.

## Introduction

Acute heart failure significantly contributes to morbidity and mortality in the western world (1). Over the last decade, hospital survival of patients with cardiogenic shock remained below 50% (2). More recent studies on cardiogenic shock focusing on standardized team-based care (3) or using mechanical circulatory support *via* an intraventricular assist device before percutaneous coronary intervention (4) showed more promising outcomes including survival rates > 70% at discharge.

Heart failure is closely linked to kidney function (5). This is also demonstrated by the fact that cardiovascular diseases are a leading cause of death for people with kidney disease (1) and that hospitalization rates for heart failure increase in patients with chronic kidney disease or albuminuria (6).

In acute heart failure, the guideline of the European Society of Cardiology recommends the use of inotropes for treatment of patients with low cardiac output and hypotension (7). Levosimendan benefits patients not only by improving myocardial contractility but may also improve overall organ perfusion *via* its vasodilatatory effect (8). Inotropes with adrenergic mechanisms on the other hand may cause tachycardia, myocardial ischemia, and even increase mortality (7, 9, 10). Some data suggest that levosimendan might be superior to dobutamine in respect to survival (11, 12).

Data on levosimendan in patients with end stage renal disease are very limited (13) and safety data is missing. Therefore, levosimendan is contraindicated in patients with an eGFR < 30 ml/min/1.73 m^2^. However, withholding levosimendan from patients with kidney dysfunction due to missing data might be ill-advised, as a number of studies suggest that levosimendan improves renal blood flow and consequently renal function (14–16). Therefore, levosimendan treatment of patients with combined heart and kidney failure requires careful consideration for each individual case.

To provide further evidence for these difficult treatment decisions, we retrospectively analyzed all patients receiving levosimendan in our intensive care unit (ICU), stratified by kidney function. Primary endpoint was a composite of known complications of inotropic agents in heart failure, including supraventricular and ventricular arrhythmias and death (7, 9, 10).

## Materials and methods

The study was designed as an investigator-initiated single-center retrospective cohort study, conducted by the standards of the Declaration of Helsinki and approved by the local ethics committee (Ethics Committee of Albert-Ludwigs-University Freiburg, file number 445/20). All patients receiving levosimendan between January 2010 and December 2019 on the intensive care units (ICUs) of the Department of Internal Medicine of the Freiburg University Medical Center were included. All data attained from the registry was anonymized to protect personal data of study subjects.

### Patient selection

Patients with a history of levosimendan treatment in the investigated time frame were identified by a computerized search for the OPS code for levosimendan: 6-004.d (OPS: German procedural classification “Operationen- und Prozedurenschlüssel”). Exclusion criteria were follow-up < 24 h following levosimendan treatment, previous levosimendan treatment < 4 weeks ago (the first dose being included in analysis), levosimendan administration started before ICU-stay and no available eGFR at day −1 or 0 of levosimendan treatment. Duplicate cases were excluded. In Germany from 2013 until 2018 levosimendan was recommended for use in eGFR > 60 ml/min/1.73 m^2^ and afterward up to >30 ml/min/1.73 m^2^ (Fachinformation Simdax, (17)). Levosimendan is not recommended in eGFR < 30 ml/min/1.73 m^2^ as of writing of this article. Patients were grouped in the following eGFR groups: >60 ml/min/1.73 m^2^, 60–30 ml/min/1.73 m^2^, <30 ml/min/1.73 m^2^.

eGFR was calculated using the “Modification of diet in renal disease”-formula [MDRD-formula, (18)]. Patients on renal replacement therapy or anuric patients (urine output of <100 ml on the day of levosimendan administration) were considered having eGFR < 30 ml/min/1.73 m^2^ regardless of eGFR calculated by MDRD-formula.

### Indication for levosimendan

Levosimendan was administered at the attending physician’s discretion. The decision to administer the drug to patients with severely reduced kidney function was made at the bedside on case-by-case basis, weighing the potential risks and benefits. Treatment decisions were not influenced by this trial. As our center does not require intensive care physicians to document cases of patients with severely reduced kidney function in which levosimendan was decided against, no control group was available.

### Clinical and laboratory baseline and follow-up

Patients’ age, body mass index (BMI), estimated glomerular filtration rate, and preexisting conditions were measured at baseline. This included a screening of all patients for preexisting chronic arrythmias which were defined as any permanent or recurring, supraventricular or ventricular arrhythmias. Preexisting cardiomyopathy was defined as myocardial disease with reduced cardiac output, either ischemic, valvular or secondary to other diseases like myocarditis or hypertension. Multimorbidity was defined as ≥ 10 preexisting conditions. Regarding ICU-admission patients were grouped to acute cardiac (e.g., cardiogenic shock, myocardial infarction), chronic cardiac (e.g., chronic cardiomyopathy, chronic valve insufficiency), extracardiac (e.g., septic shock, respiratory failure) or post resuscitation care (of any cause). Clinical parameters such as mean arterial pressure, diuresis, fluid balance, renal replacement therapy (RRT), mechanical ventilation and extracorporeal live support (ECLS) and laboratory parameters such creatinine-, urea-, electrolyte- and quick levels were analyzed over 7 days after levosimendan administration.

### Levosimendan administration

Levosimendan was administered as a continuous infusion over a period of up to 24 h. The starting rate was set at 0.025 – 0.05 μg/kg bodyweight/minute. No bolus was given. This approach is recommended in hemodynamically unstable patients including cardiogenic shock to avoid sudden hypotension (*via* the vasodilatory properties of levosimendan) and consecutive bolus therapy with vasopressors (19).

### Clinical endpoints

The predefined primary endpoint was a combination of supraventricular tachycardia (SVT), ventricular tachycardia (VT)/ventricular fibrillation (VF) and death within the first 7 days. VF was considered equivalent to VT. This endpoint was chosen according to the most relevant side effects of levosimendan (11, 12, 20). Secondary endpoints were SVT within the first 7 days, VT within the first 7 days and death within the first 7 days as well as ICU survival. Endpoints were evaluated by manual search in the electronic patient’s files including the daily documentation of physicians and nurses. Antiarrhythmic drugs class 1 and 3 given or DC shocks delivered were considered to be an equivalent to an antiarrhythmic episode. When differentiation between SVT and VT was not clear, a VT was presumed.

### Statistical analysis

Chi-Squared test, Kruskal–Wallis test, ANOVAs, Mann–Whitney test, Mantel–Cox test and uni- and multivariate logistic regression were used, as appropriate. The Shapiro–Wilk test was used for testing for normality. Unless indicated otherwise, discrete variables are shown as number and percentage, continuous variables are shown as median and interquartile range. Regression analysis was performed in two steps. First, known potential confounders of the primary endpoint were predefined and then tested in a univariate logistic regression analysis. Predefined confounders were group (as defined by eGFR), gender, age, duration of mechanical ventilation, coronary heart disease, preexisting cardiomyopathy, chronic heart failure, chronic arrhythmias, diabetes mellitus, multimorbity (as defined above), and the use of either an extracorporeal life support, an intraventricular assist device or a renal replacement therapy. Then multivariate logistic regression was performed on the predictors of the primary endpoint that had been previously identified in the univariate analysis, i.e., age, need for extracorporeal life support and renal replacement therapy.

For propensity score matching the group of patients with an eGFR of <30 ml/min/1.73 m^2^ was matched with all patients with an eGFR of >30 ml/min/1.73 m^2^. The caliper was set at 0.2 (21). Matching variables were the predictors of the primary endpoint as calculated in the multivariate logistic regression analysis.

Two-sided *p*-values < 0.05 were considered statistically significant. Data were analyzed using SPSS (Version 23, IBM Statistics, Armonk, NY, USA) and Prism (Version 9, GraphPad, San Diego, CA, USA). Registry data were checked for plausibility according to the RECORD statement (22). Data presentation follows the STROBE guidelines for reporting of observational studies (23).

### Bias

To manage bias we used the well-established KDIGO-classification of kidney disease when grouping patients by eGFR (24) and used the clearly defined outcomes of 7-day and ICU survival.

To adjust for confounding variables, we performed a multivariate logistic regression analysis including all factors with significant impact on the primary endpoint in a univariate logistic regression analysis. Factors included in the univariate logistic regression were preselected for clinical plausibility. A propensity score matching was performed using SPSS (Version 23, IBM Statistics, Armonk, NY, USA) in order to reduce bias. We predefined matching criteria to be based on items being significantly tested in the multivariate logistic regression analysis for the primary endpoint described above.

This registry by design does not include a control group of patients with severely reduced kidney function (eGFR < 30 ml/min/1.73 m^2^) not receiving levosimendan. The reason is that we expected a significant selection bias due to high treatment cost. Thus, levosimendan was presumably not given to patients with poor prognosis.

## Results

### Study population

**FIGURE 1:**
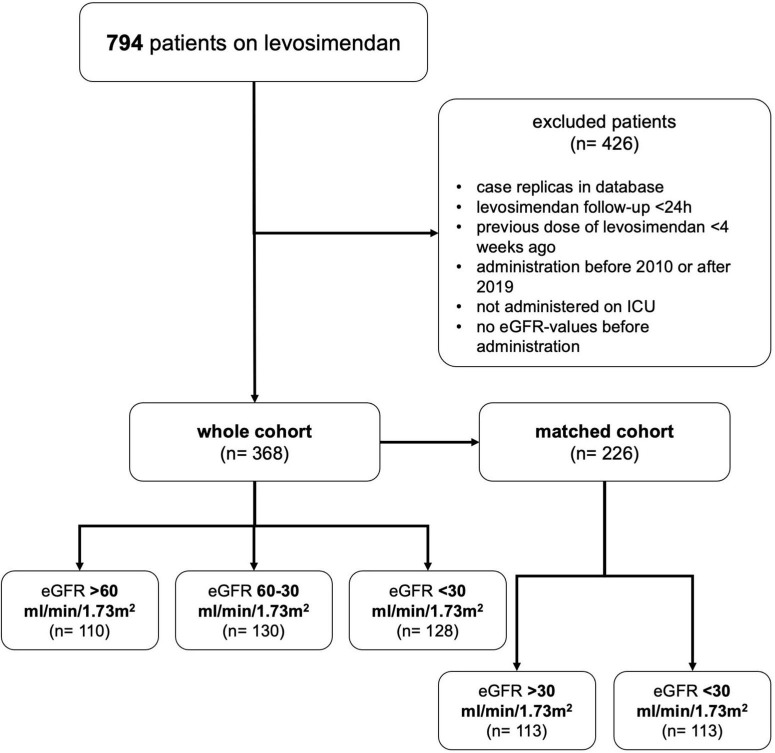
Patient inclusion. Flow chart demonstrating how patients were assigned to groups by estimated glomerular filtration rate (eGFR). Reasons for exclusion are shown.

The registry included 794 patients receiving levosimendan between January 2010 and December 2019. After exclusion of ineligible cases, 368 patients were included in this study. The most frequent reason for exclusion were case replica in our database (*n* = 180, 22.7%), levosimendan administration started before ICU-stay (*n* = 142, 17.9%) and short follow-up (<24 h; *n* = 48, 6.0%, see Figure 1).

Patients were grouped according to renal function at levosimendan administration. In the group of patients with eGFR > 60 ml/min/1.73 m^2^, median eGFR was 77.7 (69.8–94.9) ml/min/1.73 m^2^, in the eGFR 60–30 ml/min/1.73 m^2^ group median eGFR was 43.8 (37.4–52.7) ml/min/1.73 m^2^, and in the group of patients with eGFR < 30 ml/min/1.73 m^2^ median eGFR was 21.4 (16.1–27.4) ml/min/1.73 m^2^ (*p* < 0.001). Patients were predominantly male (73.6%). The median age of all patients was 63 (54–72) years but varied significantly among the groups with lower eGFR patients being of older age (*p* < 0.001). The main reasons for admission of levosimendan were acute cardiac events (55.4%) and post resuscitation care (29.3%), with acute cardiac events being more frequent in eGFR < 30 ml/min/1.73 m^2^ (66.4%, *p* = 0.008), and post resuscitation care more frequent in eGFR > 60 ml/min/1.73 m^2^ (40.0%, *p* < 0.001). Regarding preexisting conditions, chronic heart failure, chronic arrhythmias, diabetes mellitus, arterial hypertension, chronic lungs disease and chronic kidney disease and multimorbidity were more frequent in lower eGFR (all *p* < 0.05), see Table 1.

**TABLE 1:** Baseline characteristics of all patients before levosimendan administration.

Baseline characteristics	Total (*N* = 368)	eGFR > 60 ml/min/1.73 m^2^ (*N* = 110)	eGFR 60–30 ml/min/1.73 m^2^ (*N* = 130)	eGFR < 30 ml/min/1.73 m^2^ (*N* = 128)	*P*-value
Male gender	271 (73.6%)	81 (73.6%)	99 (76.2%)	91 (71.1%)	0.654
Age [year]	63 (54–72)	58 (48–70)	63.5 (54–74)	66.5 (59–75)	**<0.001**
BMI [kg/m^2^]	25.4 (24.0–28.4)	24.7 (23.4–27.5)	26.1 (24.1–29.5)	25.6 (24.2–28.5)	**0.033**
eGFR [ml/min.]	43.9 (26.1–66.2)	77.7 (69.8–94.9)	43.8 (37.4–52.7)	21.4 (16.1–27.4)	**<0.001**
Reason for admission					
Acute cardiac	204 (55.4%)	53 (48.2%)	66 (50.8%)	85 (66.4%)	**0.008**
Post resuscitation	108 (29.3%)	44 (40.0%)	42 (32.3%)	22 (17.2%)	**<0.001**
Extracardiac	33 (9.0%)	6 (5.5%)	10 (7.7%)	17 (13.3%)	0.089
Chronic cardiac	23(6.3%)	7 (6.4%)	12 (9.2%)	4 (3.1%)	0.128
Preexisting conditions					
Coronary heart disease	223 (60.6%)	60 (5.5%)	83 (63.8%)	80 (62.5%)	0.293
Cardiomyopathy	152 (41.3%)	35 (31.8%)	58 (44.6%)	59 (46.1%)	0.053
Chronic heart failure	212 (57.6%)	49 (44.5%)	78 (60.0%)	85 (66.4%)	**0.002**
Chronic arrhythmias	143 (38.9%)	19 (17.3%)	54 (41.5%)	70 (54.7%)	**<0.001**
Diabetes mellitus	100 (27.2%)	22 (20.0%)	26 (20.0%)	52 (40.6%)	**<0.001**
Hypertension	172 (46.7%)	37 (33.6%)	64 (49.2%)	71 (55.5%)	**0.003**
Chronic lung disease	63 (17.1%)	13 (11.8%)	19 (14.6%)	31 (24.2%)	**0.026**
Chronic kidney disease	90 (24.5%)	5 (4.5%)	26 (20.0%)	59 (46.1%)	**<0.001**
Chronic liver disease	20 (5.4%)	4 (3.6%)	6 (4.6%)	10 (7.8%)	0.321
Acute liver failure	37 (10.1%)	7 (6.4%)	12 (9.2%)	18 (14.1%)	0.133
MELD score	22.8 (18.6–30.9)	16.6 (13.8–18.0)	21.0 (18.7–23.8)	30.9 (27.3–32.0)	**<0.001**
Multiple chronic conditions	93 (25.3%)	9 (8.2%)	29 (22.3%)	55 (43.0%)	**<0.001**

Patient characteristics at baseline given as number of patients (percentage of patients) or as median (interquartile range). *P*-values were calculated using Chi-square-test or Kruskal–Wallis test as applicable. The *p*-value is reported in bold if the differences are statistically significant (*p* < 0.05). BMI, body mass index; eGFR, estimated glomerular filtration rate; min, minute; MELD, Model for End-Stage Liver Disease.

### Clinical and laboratory follow-up

During follow-up, groups remained segregated regarding creatinine- and urea-levels as well as diuresis and need of renal replacement therapy with lower starting eGFR corresponding with worse renal function over the follow-up period (for individual *p*-values see Table 2). Fluid balance however, did not differ significantly among groups (see Table 2). Lower starting eGFR also was associated with significantly lower mean arterial pressure (*p* = 0.045), higher lactate-levels (*p* < 0.001), higher bilirubin (*p* < 0.001) and lower quick (*p* = 0.057) on day 7 (see Table 2 and Supplementary Table 1) as surrogate parameters for circulatory and liver function. While lower eGFR at levosimendan administration correlated with lower mean arterial pressure at all points from day 0 to 7, the mean arterial pressure in each group remained constant. Norepinephrine dose within the groups was similar before and after levosimendan administration. All baseline and follow-up data are available in Table 2 and Supplementary Table 1.

**TABLE 2:** Clinical course day −1 to day 7.

	Total (d0 *N* = 368)	eGFR > 60 ml/min/1.73 m^2^ (d0 *N* = 110)	eGFR 60–30 ml/min/1.73 m^2^ (d0 *N* = 130)	eGFR < 30 ml/min/1.73 m^2^ (d0 *N* = 128)	*P*-value
**Clinical parameters**					
Mean arterial pressure [mmHg]					
d0	73 (66.5–83)	77 (68–85)	76 (69–82)	70 (64–80.3)	**<0.001**
d1	73 (66–81)	74 (67–83)	73.5 (67–82.3)	69 (63–76)	**<0.001**
d2	72 (66–83)	77 (67.3–86)	73.5 (67–83.3)	68 (63–76)	**<0.001**
d7	76 (68–87)	79 (71–89.3)	75 (66.8–87.3)	71 (67–79.5)	**0.045**
ECLS					
d0	118 (32.1%)	44 (40.0%)	41 (31.5%)	33 (25.8%)	0.063
d1	134 (36.4%)	48 (43.6%)	47 (36.2%)	39 (30.5%)	0.109
d2	121 (35.1%)	37 (35.9%)	49 (39.2%)	35 (29.9%)	0.311
d7	37 (13.8%)	6 (6.7%)	18 (18.8%)	13 (15.5%)	0.052
any time	152 (41.3%)	52 (47.3%)	57 (43.8%)	43 (33.6%)	0.078
Diuresis [ml]					
d0	1395 (515.3–2502.5)	2055 (1222.5–3017.5)	1472.5 (935–2402.5)	377.5 (50–1642.5)	**<0.001**
d1	1450 (397.5–2470)	2140 (1367–3155)	1487.5 (700–2262.5)	394 (50–1675)	**<0.001**
d2	1555 (452.5–2646.3)	2130 (1380–3200)	1775 (685–2480)	582.5 (30–1722.5)	**<0.001**
d7	1577.5 (275–2710)	1940 (1375–3450)	1465 (285–2477.5)	620 (0–1916)	**<0.001**
Fluid balance [ml]					
d0	1108 (−233.3–3340.8)	913.5 (−235.8–3948.3)	1230 (−106.8–3352.3)	1055.5 (−501.8–2916.5)	0.712
d1	145 (−862.5–1523)	−77 (−920.5–940.5)	496.5 (−625.3–1853)	−14.5 (−1304–1251.8)	**0.014**
d2	−238 (−1046–997.3)	−338 (−1059–659)	−81 (−864.5–1436)	−373 (−1657.8–1205.5)	0.081
d7	−622 (−1287.8–414.8)	−980 (−1575–66)	−254.5 (−1198.8–692.3)	−545 (−1131–356)	**0.045**
Renal replacement therapy					
d1	46 (12.5%)	0 (0.0%)	4 (3.1%)	42 (32.8%)	**<0.001**
d2	67 (19.4%)	4 (3.9%)	14 (11.2%)	49 (41.9%)	**<0.001**
d7	49 (18.2%)	3 (3.4%)	20 (20.8%)	26 (31.0%)	**<0.001**
any time	103 (28.0%)	6 (5.5%)	30 (23.1%)	67 (52.3%)	**<0.001**
Mechanical Ventilation [h]					
total (d0-7)	115 (24–234.5)	130.5 (43.8–233.8)	108.5 (43–224)	99 (0–278)	0.207
**Laboratory parameters**					
Lactate [mmol/l]					
d-1	3.1 (1.6–5.9)	2.4 (1.5–4.9)	3.3 (1.7–5.6)	3.1 (1.6–6.8)	0.147
d0	2.5 (1.5–4.5)	2.3 (1.3–4.5)	2.6 (1.6–4.4)	2.6 (1.5–5.2)	0.482
d1	1.6 (1.2–2.4)	1.5 (1.0–2.1)	1.5 (1.2–2.3)	1.8 (1.3–3.0)	**0.011**
d2	1.4 (1.0–2.3)	1.3 (0.9–1.9)	1.4 (1.0–2.1)	1.6 (1.1–2.9)	**0.003**
d7	1.3 (0.9–2.1)	1.1 (0.8–1.6)	1.3 (0.9–1.7)	1.8 (1.2–2.6)	**<0.001**
Creatinine [mg/dl]					
d-1	1.6 (1.1–2.5)	1 (0.8–1.1)	1.6 (1.4–1.9)	2.9 (2.5–3.8)	**<0.001**
d0	1.7 (1.1–2.8)	1 (0.8–1.2)	1.7 (1.3–2.2)	2.9 (2.3–3.9)	**<0.001**
d1	1.7 (1.0–2.9)	1 (0.8–1.2)	1.7 (1.3–2.6)	2.8 (2.1–3.7)	**<0.001**
d2	1.6 (1.1–2.9)	1 (0.8–1.3)	1.6 (1.2–2.7)	2.5 (1.8–3.8)	**<0.001**
d7	1.4 (1.0–2.3)	1 (0.8–1.2)	1.4 (1.1–2.3)	2.1 (1.4–3.2)	**<0.001**
Urea [mg/dl]					
d-1	67.5 (43–111.3)	38 (28–49.5)	65 (51–94.5)	122 (86.3–159.3)	**<0.001**
d0	80 (49.5–120)	44 (31–58)	75.5 (55.8–99.3)	124.5 (88–161)	**<0.001**
d1	82 (54–124.3)	49 (32–73)	80 (56.8–110)	122 (85.5–165.5)	**<0.001**
d2	80 (54–128.8)	52 (37–73)	82 (57–122)	111 (75.3–162.3)	**<0.001**
d7	76 (49–130.3)	55.5 (40.5–86.8)	83 (51–124)	101 (68–152)	**<0.001**
Sodium [mmol/l]					
d-1	137 (134–142)	138 (133–143)	138 (134–141)	137 (134–140)	0.286
d0	139 (135–144)	140 (136–145)	141 (135–144)	138 (134–142)	**0.006**
d1	140 (135–144)	140 (136–146)	140 (135–145)	139 (134–142)	**0.001**
d2	140 (136–144)	142 (136–146)	141 (136–146)	139 (135–143)	**0.016**
d7	141 (136–146)	141 (137–146)	142 (136–147)	140 (136–144)	0.204
Potassium [mmol/l]					
d-1	4.4 (4.0–5.0)	4.2 (3.8–4.5)	4.4 (3.9-4.9)	4.7 (4.1–5.3)	**<0.001**
d0	4.4 (4.1–4.8)	4.3 (4.0–4.6)	4.3 (4.1–4.8)	4.5 (4.1–4.9)	**0.001**
d1	4.4 (4.1–4.7)	4.4 (4.1–4.8)	4.4 (4.1–4.7)	4.4 (4.1–4.7)	0.905
d2	4.4 (4.2–4.8)	4.3 (4.1–4.7)	4.5 (4.3–4.9)	4.4 (4.1–4.9)	0.042
d7	4.4 (4.1–4.7)	4.2 (4.0–4.5)	4.4 (4.1–4.8)	4.4 (4.2–4.8)	**0.002**
Quick [%]					
d-1	57 (40–76)	72 (53–83)	57 (42–72)	46 (32–66)	**<0.001**
d0	58 (39–77)	70 (50–85)	58 (41–77)	46 (30–67)	**<0.001**
d1	60 (43–82)	76 (54–92)	62 (48–83)	50 (37–67)	**<0.001**
d2	66 (46–87)	76 (57–97)	70 (50–84)	53 (41–71)	**<0.001**
d7	75 (58–88)	76 (59–95)	78 (63–87)	69 (51–82)	**0.057**

### Clinical endpoints

**FIGURE 2:**
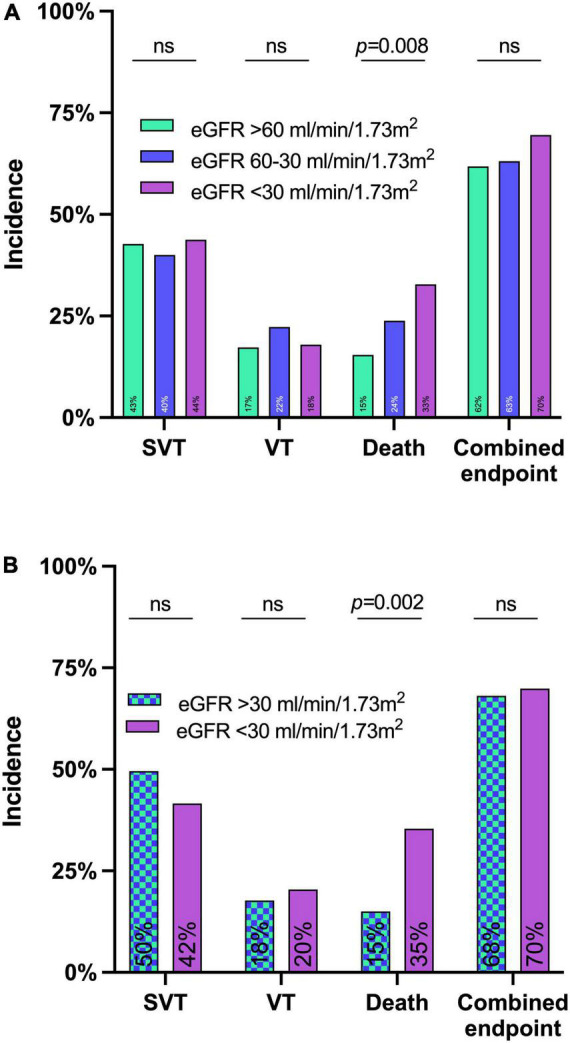
Incidence of the primary and secondary endpoints until day 7 after levosimendan administration in the whole cohort **(A)** and the matched cohort **(B)**. eGFR before levosimendan administration had no significant impact on the combined endpoint of either death or supraventricular tachycardias (SVT) or ventricular tachycardias (VT) until day 7 in neither the whole cohort in panel **(A)** nor the matched cohort in panel **(B)**.

**FIGURE 3:**
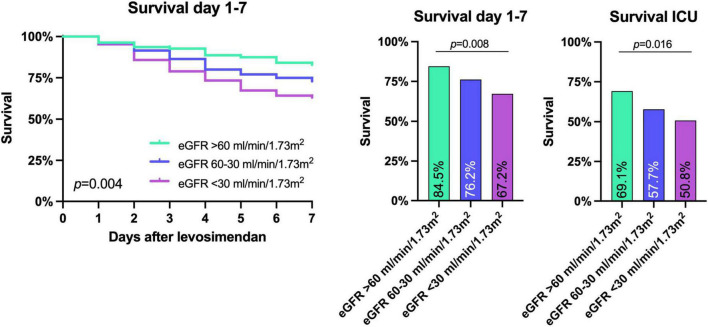
Survival until day 7 after levosimendan administration and intensive care unit (ICU) survival. Lower eGFR before levosimendan administration corresponded to both a lower 7 day and ICU survival as shown by Kaplan Meier survival curves and Chi-Square analysis. *P*-values are shown in the figure.

The primary combined endpoint was reached in each group at similar rates (61.8, 63.1, and 69.5% in patients with eGFR > 60 ml/min/1.73 m^2^, 60–30 ml/min/1.73 m^2^, and <30 ml/min/1.73 m^2^, respectively, *p* = 0.396), see Figure 2. The 7-day survival was significantly lower in lower eGFR groups (7 d survival: 84.5% of patients survived in eGFR > 60 ml/min/1.73 m^2^, 76.2% in eGFR 60–30 ml/min/1.73 m^2^, and 67.2% in eGFR < 30 ml/min/1.73 m^2^, respectively, *p* < 0.01). ICU-survival showed a similar pattern with significantly lower survival in patients with decreased kidney function (*p* < 0.02), see Figure 2, 3 and Table 3A.

**TABLE 3:** Primary and secondary endpoints in the whole cohort **(A)** and the matched cohort **(B)**.

A: Whole cohort	Total (*N* = 368)	eGFR > 60 ml/min/1.73 m^2^ (*N* = 110)	eGFR 60–30 ml/min/1.73 m^2^ (*N* = 130)	eGFR < 30 ml/min/1.73 m^2^ (*N* = 128)	*P*-value
Endpoint					
Combined endpoint	239 (64.9%)	68 (61.8%)	82 (63.1%)	89 (69.5%)	0.396
Supraventricular tachycardias	155 (42.1%)	47 (42.7%)	52 (40.0%)	56 (43.8%)	0.821
Ventricular tachycardias	71 (19.3%)	19 (17.3%)	29 (22.3%)	23 (18.0%)	0.551
Mortality d0-7	90 (24.5%)	17 (15.5%)	31 (23.8%)	42 (32.8%)	**0.008**
ICU Mortality	152 (41.3%)	34 (30.9%)	55 (42.3%)	63 (49.2%)	**0.016**
**B: Matched cohort**			**eGFR > 30** **ml/min/1.73 m^2^** **(*N* = 113)**	**eGFR < 30** **ml/min/1.73 m^2^** **(*N* = 113)**	***P*-value**

Endpoint					
Combined endpoint			77 (68.1%)	79 (69.9%)	0.886
Supraventricular tachycardias			56 (49.6%)	47 (41.6%)	0.285
Ventricular tachycardias			20 (17.7%)	23 (20.4%)	0.735
Mortality d0-7			17 (15.0%)	40 (35.4%)	**0.001**

Endpoint data given as number of patients (percentage of patients). *P*-values were calculated using Chi-square-test and Fisher’s exact test. The *p*-value is reported in bold if the differences are statistically significant (*p* < 0.05). All endpoints but ICU mortality include data for d0-7 only. The propensity score matching was based on the predictors of outcome (age, extracorporeal life support at any time, renal replacement therapy at any time) identified in the regression analysis. eGFR, estimated glomerular filtration rate; min, minute; ICU, intensive care unit.

When dividing the group of patients with eGFR < 30 ml/min/1.73 m^2^ in those with and without RRT before levosimendan administration, endpoints were statistically similar, see Supplementary Table 2.

### Confounders

Age, preexisting cardiomyopathy, chronic heart failure and necessity for ELCS and RRT during day 1-7 significantly impacted the primary endpoint in a univariate regression model, see Table 4. The eGFR before levosimendan administration however, had no significant impact on the primary endpoint (*p* = 0.206). A multivariate logistic regression model confirmed age (*p* < 0.02), and ECLS (*p* < 0.001) or RRT (*p* = 0.028) during day 1-7 as predictors for the primary endpoint. Renal function did not affect the primary endpoint (*p* = 0.835), see Table 4.

**TABLE 4:** Predictors of the primary endpoint.

	Univariate logistic regression analysis			Multivariate logistic regression analysis		
Variable	Hazard ratio	95% confidence interval	*P*-value	Hazard ratio	95% confidence interval	*P*-value
Group	1.188	(0.910 to 1.553)	0.206	1.036	(0.743 to 1.444)	0.835
Male gender	1.130	(0.697 to 1.830)	0.621			
Age [year]	1.018	(1.003 to 1.033)	**0.018**	1.021	(1.004 to 1.038)	**0.014**
IMV duration [hour]	1.000	(1.000 to 1.001)	0.329			
CHD	1.427	(0.923 to 2.206)	0.109			
Cardiomyopathy	0.537	(0.347 to 0.829)	**0.005**	0.953	(0.521 to 1.744)	0.877
Chronic heart failure	0.502	(0.321 to 0.787)	**0.003**	0.739	(0.396 to 1.379)	0.342
Chronic arrhythmias	1.297	(0.832 to 2.023)	0.251			
Diabetes mellitus	1.003	(0.620 to 1.624)	0.989			
Multimorbid	0.634	(0.392 to 1.027)	0.064			
ECLS	2.725	(1.709 to 4.345)	**<0.001**	2.636	(1.573 to 4.418)	**<0.001**
PVAD	1.906	(0.907 to 4.006)	0.089			
RRT	2.160	(1.286 to 3.630)	**0.004**	1.949	(1.076 to 3.53)	**0.028**

Predefined potential confounders for the primary endpoint (supraventricular-, ventricular tachycardia and death within 7 days after administration of levosimendan) were tested in an univariate and multivariate logistic regression analysis. “Group” as defined by the three eGFR cohorts. Only age and need for RRT or ECLS day 1-7 predicted the combined endpoint while baseline kidney function did not. IMV, invasive mechanical ventilation; ELCS, extracorporeal life support; PVAD, percutaneous ventricular assist device; RRT, renal replacement therapy.

### Propensity score matching

A propensity score matching was computed using age, ECLS, and RRT during day 1-7 as factors as identified by multivariate logistic regression. The matched cohort now consisted of 113 patients with an eGFR > 30 ml/min/1.73 m^2^ and 113/128 patients with eGFR < 30 ml/min/1.73 m^2^ (see Supplementary Table 3). The matched cohort showed no differences regarding the primary endpoint (68.1 vs. 69.9%; *p* = 0.886) while 7-day mortality remained higher in eGFR < 30 ml/min/1.73 m^2^ (15.0 vs. 35.4%; *p* = 0.001) – see Table 3B.

## Discussion

In this retrospective registry of patients receiving levosimendan as rescue therapy in cardiogenic shock, the primary combined endpoint of death within 7 days, supraventricular and ventricular tachycardia was reached at similar rates in patients with reduced and normal kidney function.

In patients with severely reduced kidney function, levosimendan is not recommended since data are missing. This is a common problem in clinical trials since patients with kidney disease are underrepresented (25). Even though acute and chronic kidney disease is present in 20–60% of patients with heart failure (26–28), patients with kidney disease are frequently excluded from trials (29, 30). Many arguments exist for excluding these patients including the fact that prognosis of patients with kidney disease is significantly worse (31). Data from patients with good kidney function however, cannot be easily extrapolated to those with kidney dysfunction (32). Therefore, uncertainty remains for this important patient group.

There are data on levosimendan in end-stage renal disease. Data on 24 patients end stage renal disease suggest that half-life of levosimendan is prolonged 1.5-fold and peak plasma concentration is increased 2-fold (33). The clinical significance of these findings is unclear as no serious side effects were documented (33).

A recent study using the Chang Gung Research Database from Taiwan on 52 patients with eGFR < 30 ml/min/1.73 m^2^ receiving levosimendan showed similar outcomes compared to 374 patients receiving dobutamine (13). Similar results are reported from 25 patients with end-stage renal disease undergoing coronary artery bypass grafting who received levosimendan showing similar side effects compared to 33 patients on placebo (34). To the best of our knowledge no other study has shown an increase in side effects of levosimendan when used in eGFR < 30 ml/min/1.73 m^2^ either.

On the other hand, many data suggest a potential benefit of levosimendan in kidney failure including reduced ischemia/reperfusion injuries in animals (35). In the perioperative setting, levosimendan might lower the incidence of acute kidney injury as suggested by a meta-analysis including 529 patients (36). A finding which might be at least in part explained by the finding that levosimendan increases renal blood flow through renal vasodilatation (16). On the other hand, bolus-free levosimendan administration may have prevented significant hypotension reported in patients after administration of a bolus of the inodilatator levosimendan (19). These findings fit well into data reported here, showing stable kidney function after levosimendan in the context of acute heart failure, a condition known to affect kidney function normally negatively (37).

In this study, the primary endpoint of SVT, VT, or death within 7 days of levosimendan application did not vary among the predefined groups of kidney function. Nevertheless, lower eGFR was associated with a lower 7-day and ICU survival. This seems plausible, as lower eGFR was also associated with advanced age, chronic heart failure, arrhythmias, arterial hypertension, diabetes mellitus and chronic lung disease in our dataset, all of which are known predictors of mortality (38–41).

Despite these considerable differences in baseline characteristics, uni- and multivariate logistic regressions analysis identified only age and the need of either ECLS or RRT as predictors of the combined endpoint and not the baseline eGFR. In addition, the individual endpoints of SVTs and VTs were not more frequent in lower eGFR suggesting that levosimendan could be used safely in severely reduced kidney function. These findings were strengthened by coherent results in the propensity score matched cohort.

## Limitations

Several limitations have to be considered when interpreting the presented results: First, this is a retrospective registry study including only patients receiving levosimendan, as indicated by discretion of the treating physician. As patients with a high risk of complications by levosimendan including those with ventricular arrhythmias or those with high doses of vasopressors probably were not considered candidates for an inodilator, a selection bias is likely. Second, complications are notoriously underreported in retrospective trials (42). Even though data acquisition was performed diligently, we have to presume that not every arrhythmic episode was documented. This methodical error should affect all three groups. Third, groups were divided by the calculated eGFR, anuria and renal replacement therapy at the time of levosimendan administration. We cannot rule out that a more precise method of measuring kidney function would have changed group allocation (43). Group allocation according to kidney function however, remained stable over the study; therefore, the number of incorrectly classified patients might be low. Fourth, patients were only followed during their stay on ICU. We cannot exclude that potential life-threatening complications caused by levosimendan were detected and handled adequately therefore not impacting the combined endpoint. If levosimendan can be administered to patients with end-stage kidney function outside an ICU cannot be answered by this registry, though we did not find an increase in arrhythmias in our data. Fifth, we controlled for lack of a randomized control group by propensity score matching. This process even though valuable in retrospective data analysis, cannot replace the benefits of a prospective randomized and controlled trial. Last, we could not include data from echocardiographic exams since no structured documentation of transthoracic or transesophageal echocardiography were available in the studied data sources.

## Conclusion

The primary combined endpoint of supraventricular-, and ventricular tachycardia as well as death by day 7 was reached at a similar rate in patients with lightly, moderately and severely reduced kidney function. Prospective randomized trials are warranted in order to clarify if levosimendan can be used safely in severely reduced kidney function.

## Data availability statement

The raw data supporting the conclusions of this article will be made available by the authors, without undue reservation.

## Ethics statement

The studies involving human participants were reviewed and approved by the Ethics Committee of Albert-Ludwigs-University Freiburg/Germany. Written informed consent for participation was not required for this study in accordance with the national legislation and the institutional requirements.

## Author contributions

FR, AB, ToW, and DS planned the study. FR and AB screened all patients and included data in registry. FR, AB, XB, ThW, AS, and DS analyzed the data and prepared the figures. FR, AB, and DS wrote the main manuscript text. XB, ThW, AS, and ToW critically reviewed the data. All authors reviewed the manuscript and approved the submitted version.
