# Supplementary material for: Levosimendan in acute heart failure with severely reduced kidney function, a propensity score matched registry study
Source: Front Cardiovasc Med. 2022 Oct 20;9:1027727. doi: 10.3389/fcvm.2022.1027727 (PMC9631470; doi:10.3389/fcvm.2022.1027727)
Supplement: Supplementary file 1 [file Data_Sheet_1.docx]

**Supp. material**

**Supp. Table 1** Clinical course day -1 to day 7

Follow-up data given as number of patients (percentage of patients) or as median (interquartile range). P-values were calculated using Chi-square-test, Kruskal-Wallis or 1way-ANOVA test as applicable. The p-value is reported in bold if the differences are statistically significant (p<0.05). Abbreviations: eGFR: estimated glomerular filtration rate, min.: minute, d: day, ECLS: extracorporeal life support, PVAD: percutaneous ventricular assist device

|  | N | Total | eGFR >60  ml/min/1.73m^2^ | eGFR 60-30  ml/min/1.73m^2^ | eGFR <30  ml/min/1.73m^2^ | p-value |
| --- | --- | --- | --- | --- | --- | --- |
| **Clinical parameters** | |  |  |  |  |  |
| Heart rate [1/minute] | |  |  |  |  |  |
| d0 | 368:110:130:128 | 87 (71 - 105) | 92.5 (75.5 - 108) | 81 (67.5 - 102) | 83.5 (71 - 101) | **0.049** |
| d1 | 368:110:130:128 | 90 (75 - 107) | 96 (81 - 108.8) | 89 (72 - 105) | 87 (75 - 107) | 0.051 |
| d2 | 325:99:115:111 | 90 (78 - 108) | 97 (79 - 108.5) | 89 (80 - 104) | 89 (75 - 109.5) | 0.752 |
| d7 | 187:64:68:55 | 84 (73 - 99) | 89.5 (73.5 - 99.3) | 86 (72.8 - 99) | 82 (73.5 - 95) | 0.760 |
| Mean arterial pressure [mmHg] | |  |  |  |  |  |
| d0 | 367:110:129:128 | 73 (66.5 - 83) | 77 (68 - 85) | 76 (69 - 82) | 70 (64 - 80.3) | **<0.001** |
| d1 | 365:109:128:128 | 73 (66 - 81) | 74 (67 - 83) | 73.5 (67 - 82.3) | 69 (63 - 76) | **<0.001** |
| d2 | 321:98:112:111 | 72 (66 - 83) | 77 (67.3 - 86) | 73.5 (67 - 83.3) | 68 (63 - 76) | **<0.001** |
| d7 | 187:64:68:55 | 76 (68 - 87) | 79 (71 - 89.3) | 75 (66.8 - 87.3) | 71 (67 - 79.5) | **0.045** |
| Norepinephrine [µg/h] | |  |  |  |  |  |
| d0 | 368:110:130:128 | 600 (0 - 1800) | 560 (0 - 1700) | 480 (0 - 1800) | 700 (0 - 2200) | 0,144 |
| d1 | 368:110:130:128 | 480 (0 - 1700) | 540 (0 - 1780) | 360 (0 - 1400) | 600 (0 - 1880) | 0,377 |
| d2 | 325:99:115:111 | 260 (0 - 1200) | 200 (0 - 1200) | 200 (0 - 1000) | 400 (0 - 1300) | 0,241 |
| d7 | 187:64:68:55 | 0 (0 - 500) | 0 (0 - 480) | 0 (0 - 400) | 120 (0 - 600) | 0,304 |
| Diuresis [ml] | |  |  |  |  |  |
| d0 | 368:110:130:128 | 1395 (515.3 - 2502.5) | 2055 (1222.5 - 3017.5) | 1472.5 (935 - 2402.5) | 377.5 (50 - 1642.5) | **<0.001** |
| d1 | 343:103:122:118 | 1450 (397.5 - 2470) | 2140 (1367 - 3155) | 1487.5 (700 - 2262.5) | 394 (50 - 1675) | **<0.001** |
| d2 | 298:97:103:98 | 1555 (452.5 - 2646.3) | 2130 (1380 - 3200) | 1775 (685 - 2480) | 582.5 (30 - 1722.5) | **<0.001** |
| d7 | 174:59:60:55 | 1577.5 (275 - 2710) | 1940 (1375 - 3450) | 1465 (285 - 2477.5) | 620 (0 - 1916) | **<0.001** |
| Fluid balance [ml] | |  |  |  |  |  |
| d0 | 368:110:130:128 | 1108 (-233.3 - 3340.8) | 913.5 (-235.8 - 3948.3) | 1230 (-106.8 - 3352.3) | 1055.5 (-501.8 - 2916.5) | 0.712 |
| d1 | 343:103:122:118 | 145 (-862.5 - 1523) | -77 (-920.5 - 940.5) | 496.5 (-625.3 - 1853) | -14.5 (-1304 - 1251.8) | **0.014** |
| d2 | 298:97:103:98 | -238 (-1046 - 997.3) | -338 (-1059 - 659) | -81 (-864.5 - 1436) | -373 (-1657.8 - 1205.5) | 0.081 |
| d7 | 174:59:60:55 | -622 (-1287.8 - 414.8) | -980 (-1575 - 66) | -254.5 (-1198.8 - 692.3) | -545 (-1131 - 356) | **0.045** |
| ECLS | |  |  |  |  |  |
| d0 | 368:110:130:128 | 118 (32.1%) | 44 (40.0%) | 41 (31.5%) | 33 (25.8%) | 0.063 |
| d1 | 368:110:130:128 | 134 (36.4%) | 48 (43.6%) | 47 (36.2%) | 39 (30.5%) | 0.109 |
| d2 | 345:103:125:117 | 121 (35.1%) | 37 (35.9%) | 49 (39.2%) | 35 (29.9%) | 0.311 |
| d7 | 269:89:96:84 | 37 (13.8%) | 6 (6.7%) | 18 (18.8%) | 13 (15.5%) | 0.052 |
| any time | 368:110:130:128 | 152 (41.3%) | 52 (47.3%) | 57 (43.8%) | 43 (33.6%) | 0.078 |
| PVAD |  |  |  |  |  |  |
| d0 | 368:110:130:128 | 22 (6.0%) | 5 (4.5%) | 12 (9.2%) | 5 (3.9%) | 0.148 |
| d1 | 368:110:130:128 | 37 (10.1%) | 12 (10.9%) | 16 (12.3%) | 9 (7.0%) | 0.348 |
| d2 | 345:103:125:117 | 35 (10.1%) | 12 (11.7%) | 16 (12.8%) | 7 (6.0%) | 0.179 |
| d7 | 269:89:96:84 | 11 (4.1%) | 3 (3.4%) | 5 (5.2%) | 3 (3.6%) | 0.786 |
| any time | 368:110:130:128 | 43 (11.7%) | 14 (12.7%) | 19 (14.6%) | 10 (7.8%) | 0.217 |
| Renal replacement therapy | |  |  |  |  |  |
| d0 | 368:110:130:128 | 34 (9.2%) | 0 (0.0%) | 0 (0.0%) | 34 (26.7%) | **<0.001** |
| d1 | 368:110:130:128 | 46 (12.5%) | 0 (0.0%) | 4 (3.1%) | 42 (32.8%) | **<0.001** |
| d2 | 345:103:125:117 | 67 (19.4%) | 4 (3.9%) | 14 (11.2%) | 49 (41.9%) | **<0.001** |
| d7 | 269:89:96:84 | 49 (18.2%) | 3 (3.4%) | 20 (20.8%) | 26 (31.0%) | **<0.001** |
| any time | 368:110:130:128 | 103 (28.0%) | 6 (5.5%) | 30 (23.1%) | 67 (52.3%) | **<0.001** |
| Mechanical Ventilation [h] | |  |  |  |  |  |
| total (d0-7) | 367:110:130:127 | 115 (24 - 234.5) | 130.5 (43.8 - 233.8) | 108.5 (43 - 224) | 99 (0 - 278) | 0.207 |
| **Laboratory parameters** | |  |  |  |  |  |
| Hemoglobin [g/dl] | |  |  |  |  |  |
| d-1 | 359:109:127:123 | 11.6 (9.5 - 13.6) | 11.6 (9.7 - 13.7) | 11.7 (9.8 - 13.8) | 11.3 (9.1 - 13.3) | 0.288 |
| d0 | 366:110:130:126 | 10.6 (8.7 - 12.6) | 10.6 (8.9 - 13) | 10.7 (8.7 - 12.5) | 10.7 (8.6 - 12.5) | 0.902 |
| d1 | 367:110:129:128 | 9.7 (8.5 - 11.5) | 9.6 (8.5 - 11.4) | 9.6 (8.5 - 11.5) | 10 (8.4 - 11.6) | 0.942 |
| d2 | 353:106:124:123 | 9.1 (8.3 - 10.7) | 9 (8.3 - 10.7) | 9.1 (8.3 - 10.7) | 9.3 (8.2 - 10.7) | 0.988 |
| d7 | 314:96:111:107 | 8.8 (8.1 - 10.2) | 9 (8.3 - 10.6) | 8.8 (8.2 - 10.1) | 8.6 (7.8 - 10.1) | 0.154 |
| Lactate [mmol/l] | |  |  |  |  |  |
| d-1 | 356:107:126:123 | 3.1 (1.6 - 5.9) | 2.4 (1.5 - 4.9) | 3.3 (1.7 - 5.6) | 3.1 (1.6 - 6.8) | 0.147 |
| d0 | 365:109:130:126 | 2.5 (1.5 - 4.5) | 2.3 (1.3 - 4.5) | 2.6 (1.6 - 4.4) | 2.6 (1.5 - 5.2) | 0.482 |
| d1 | 363:107:129:127 | 1.6 (1.2 - 2.4) | 1.5 (1 - 2.1) | 1.5 (1.2 - 2.3) | 1.8 (1.3 - 3) | **0.011** |
| d2 | 346:103:121:122 | 1.4 (1 - 2.3) | 1.3 (0.9 - 1.9) | 1.4 (1 - 2.1) | 1.6 (1.1 - 2.9) | **0.003** |
| d7 | 309:95:109:105 | 1.3 (0.9 - 2.1) | 1.1 (0.8 - 1.6) | 1.3 (0.9 - 1.7) | 1.8 (1.2 - 2.6) | **<0.001** |
| Urea [mg/dl] | |  |  |  |  |  |
| d-1 | 340:103:115:122 | 67.5 (43 - 111.3) | 38 (28 - 49.5) | 65 (51 - 94.5) | 122 (86.3 - 159.3) | **<0.001** |
| d0 | 331:93:120:118 | 80 (49.5 - 120) | 44 (31 - 58) | 75.5 (55.8 - 99.3) | 124.5 (88 - 161) | **<0.001** |
| d1 | 306:87:108:111 | 82 (54 - 124.3) | 49 (32 - 73) | 80 (56.8 - 110) | 122 (85.5 - 165.5) | **<0.001** |
| d2 | 294:81:109:104 | 80 (54 - 128.8) | 52 (37 - 73) | 82 (57 - 122) | 111 (75.3 - 162.3) | **<0.001** |
| d7 | 268:86:93:89 | 76 (49 - 130.3) | 55.5 (40.5 - 86.8) | 83 (51 - 124) | 101 (68 - 152) | **<0.001** |
| Creatinine [mg/dl] | |  |  |  |  |  |
| d-1 | 350:107:119:124 | 1.6 (1.1 - 2.5) | 1 (0.8 - 1.1) | 1.6 (1.4 - 1.9) | 2.9 (2.5 - 3.8) | **<0.001** |
| d0 | 359:106:130:123 | 1.7 (1.1 - 2.8) | 1 (0.8 - 1.2) | 1.7 (1.3 - 2.2) | 2.9 (2.3 - 3.9) | **<0.001** |
| d1 | 340:102:122:116 | 1.7 (1 - 2.9) | 1 (0.8 - 1.2) | 1.7 (1.3 - 2.6) | 2.8 (2.1 - 3.7) | **<0.001** |
| d2 | 316:95:114:107 | 1.6 (1.1 - 2.9) | 1 (0.8 - 1.3) | 1.6 (1.2 - 2.7) | 2.5 (1.8 - 3.8) | **<0.001** |
| d7 | 291:96:104:91 | 1.4 (1 - 2.3) | 1 (0.8 - 1.2) | 1.4 (1.1 - 2.3) | 2.1 (1.4 - 3.2) | **<0.001** |
| eGFR [ml/min/1.73m^2^] | |  |  |  |  |  |
| d-1 | 350:107:119:124 | 43.9 (26.1 - 66.2) | 77.7 (69.8 - 94.9) | 43.8 (37.4 - 52.7) | 21.4 (16.1 - 27.4) | **<0.001** |
| d0 | 359:106:130:123 | 40.4 (23.9 - 66.6) | 80.4 (62.3 - 103.5) | 42.8 (31.6 - 53.3) | 22 (15.5 - 27.6) | **<0.001** |
| d1 | 340:102:122:116 | 40.4 (22 - 70.3) | 80.5 (62.4 - 105.3) | 40.9 (23.5 - 59.9) | 21.3 (15.5 - 31.1) | **<0.001** |
| d2 | 316:95:114:107 | 41.3 (23.7 - 69.4) | 76.3 (54.6 - 101.1) | 40.4 (25.3 - 62) | 26.5 (15.2 - 36.3) | **<0.001** |
| d7 | 291:96:104:91 | 53.8 (29.8 - 80.5) | 82 (60.9 - 103.5) | 51.1 (29.7 - 66.2) | 33.6 (19.1 - 50.5) | **<0.001** |
| Sodium [mmol/l] | |  |  |  |  |  |
| d-1 | 355:107:125:123 | 137 (134 - 142) | 138 (133 - 143) | 138 (134 - 141) | 137 (134 - 140) | 0.286 |
| d0 | 366:110:129:127 | 139 (135 - 144) | 140 (136 - 145) | 141 (135 - 144) | 138 (134 - 142) | **0.006** |
| d1 | 364:109:128:127 | 140 (135 - 144) | 140 (136 - 146) | 140 (135 - 145) | 139 (134 - 142) | **0.001** |
| d2 | 347:106:121:120 | 140 (136 - 144) | 142 (136 - 146) | 141 (136 - 146) | 139 (135 - 143) | **0.016** |
| d7 | 313:95:110:108 | 141 (136 - 146) | 141 (137 - 146) | 142 (136 - 147) | 140 (136 - 144) | 0.204 |
| Potassium [mmol/l] | |  |  |  |  |  |
| d-1 | 360:109:127:124 | 4.4 (4 - 5) | 4.2 (3.8 - 4.5) | 4.4 (3.9 - 4.9) | 4.7 (4.1 - 5.3) | **<0.001** |
| d0 | 367:110:130:127 | 4.4 (4.1 - 4.8) | 4.3 (4 - 4.6) | 4.3 (4.1 - 4.8) | 4.5 (4.1 - 4.9) | **0.001** |
| d1 | 368:110:130:128 | 4.4 (4.1 - 4.7) | 4.4 (4.1 - 4.8) | 4.4 (4.1 - 4.7) | 4.4 (4.1 - 4.7) | 0.905 |
| d2 | 354:107:124:123 | 4.4 (4.2 - 4.8) | 4.3 (4.1 - 4.7) | 4.5 (4.3 - 4.9) | 4.4 (4.1 - 4.9) | 0.042 |
| d7 | 314:96:110:108 | 4.4 (4.1 - 4.7) | 4.2 (4 - 4.5) | 4.4 (4.1 - 4.8) | 4.4 (4.2 - 4.8) | **0.002** |
| Calcium [mmol/l] | |  |  |  |  |  |
| d-1 | 360:108:127:125 | 1.1 (1.1 - 1.2) | 1.2 (1.1 - 1.2) | 1.1 (1.1 - 1.2) | 1.1 (1.1 - 1.2) | 0.044 |
| d0 | 367:110:129:128 | 1.1 (1.1 - 1.2) | 1.1 (1.1 - 1.2) | 1.1 (1.1 - 1.2) | 1.1 (1 - 1.1) | **<0.001** |
| d1 | 367:110:129:128 | 1.1 (1.1 - 1.2) | 1.1 (1.1 - 1.2) | 1.1 (1.1 - 1.2) | 1.1 (1.1 - 1.2) | 0.162 |
| d2 | 353:106:124:123 | 1.1 (1.1 - 1.2) | 1.2 (1.1 - 1.2) | 1.1 (1.1 - 1.2) | 1.1 (1.1 - 1.2) | 0.054 |
| d7 | 315:97:110:108 | 1.1 (1.1 - 1.2) | 1.2 (1.1 - 1.2) | 1.1 (1.1 - 1.2) | 1.1 (1.1 - 1.2) | **0.002** |
| Chloride [mmol/l] | |  |  |  |  |  |
| d-1 | 279:78:96:105 | 106 (101 - 110) | 106 (101 - 110) | 106 (101 - 110) | 104 (100 - 110) | 0.202 |
| d0 | 296:82:105:109 | 107 (103 - 111) | 108 (104 - 112) | 108 (104 - 112) | 105 (101 - 109) | **0.002** |
| d1 | 293:81:103:109 | 107 (102 - 112) | 109 (104 - 114) | 108 (104 - 114) | 105 (101 - 109) | **<0.001** |
| d2 | 279:79:97:103 | 107 (103 - 112) | 108 (104 - 113) | 108 (103 - 113) | 106 (103 - 110) | **0.029** |
| d7 | 261:76:92:93 | 109 (105 - 114) | 111 (105 - 115) | 110 (105 - 115) | 108 (104 - 112) | 0.304 |
| pH |  |  |  |  |  |  |
| d-1 | 361:109:127:125 | 7.36 (7.27 - 7.43) | 7.38 (7.25 - 7.45) | 7.36 (7.27 - 7.43) | 7.35 (7.27 - 7.41) | 0.555 |
| d0 | 368:110:130:128 | 7.38 (7.32 - 7.44) | 7.39 (7.32 - 7.46) | 7.39 (7.31 - 7.44) | 7.37 (7.32 - 7.43) | 0.239 |
| d1 | 368:110:130:128 | 7.4 (7.35 - 7.45) | 7.41 (7.35 - 7.46) | 7.4 (7.36 - 7.44) | 7.39 (7.35 - 7.44) | 0.190 |
| d2 | 353:107:124:122 | 7.41 (7.34 - 7.45) | 7.4 (7.34 - 7.45) | 7.41 (7.35 - 7.45) | 7.4 (7.33 - 7.44) | 0.766 |
| d7 | 316:97:111:108 | 7.42 (7.37 - 7.46) | 7.42 (7.4 - 7.47) | 7.42 (7.37 - 7.46) | 7.41 (7.34 - 7.46) | 0.175 |
| SBC [mmol/l] | |  |  |  |  |  |
| d-1 | 351:106:124:121 | 21 (18.3 - 23.8) | 21.5 (18.6 - 25.1) | 21.1 (18.6 - 23.8) | 20.5 (18 - 22.7) | 0.096 |
| d0 | 365:109:129:127 | 22 (19.2 - 24.8) | 22.7 (20.4 - 25.1) | 21.8 (19.3 - 25) | 21.5 (18.9 - 24) | 0.074 |
| d1 | 361:109:127:125 | 23.5 (21.3 - 25.4) | 24.3 (21.8 - 26.4) | 23.6 (21.8 - 25.4) | 22.6 (21.3 - 25.1) | **0.040** |
| d2 | 350:105:124:121 | 23.7 (21.5 - 25.5) | 24.1 (22.3 - 26) | 23.7 (22.1 - 25.3) | 22.9 (20.5 - 25.3) | **0.034** |
| d7 | 317:97:112:108 | 24.2 (22 - 26.3) | 24.5 (22.8 - 26.7) | 24.1 (22 - 26.2) | 24.2 (21.4 - 26.2) | 0.166 |
| Bilirubin [mg/dl] | |  |  |  |  |  |
| d-1 | 328:100:112:116 | 1 (0.5 - 1.8) | 0.8 (0.5 - 1.2) | 0.9 (0.4 - 1.5) | 1.4 (0.7 - 2.2) | **<0.001** |
| d0 | 348:103:122:123 | 1.1 (0.6 - 2) | 0.9 (0.5 - 1.3) | 1.1 (0.6 - 2.2) | 1.5 (0.9 - 2.5) | **<0.001** |
| d1 | 334:101:114:119 | 1.2 (0.6 - 2.2) | 0.8 (0.5 - 1.7) | 1.2 (0.5 - 2.1) | 1.8 (0.9 - 3) | **<0.001** |
| d2 | 304:87:104:113 | 1.2 (0.6 - 2.6) | 0.8 (0.5 - 1.3) | 1.3 (0.7 - 2.4) | 1.8 (0.8 - 3.6) | **<0.001** |
| d7 | 266:81:90:95 | 1 (0.5 - 2.4) | 0.8 (0.4 - 1.5) | 1 (0.5 - 2.1) | 1.5 (0.7 - 3.5) | **<0.001** |
| Quick [%] |  |  |  |  |  |  |
| d-1 | 343:104:118:121 | 57 (40 - 76) | 72 (53 - 83) | 57 (42 - 72) | 46 (32 - 66) | **<0.001** |
| d0 | 357:105:125:127 | 58 (39 - 77) | 70 (50 - 85) | 58 (41 - 77) | 46 (30 - 67) | **<0.001** |
| d1 | 327:97:115:115 | 60 (43 - 82) | 76 (54 - 92) | 62 (48 - 83) | 50 (37 - 67) | **<0.001** |
| d2 | 298:86:109:103 | 66 (46 - 87) | 76 (57 - 97) | 70 (50 - 84) | 53 (41 - 71) | **<0.001** |
| d7 | 257:79:91:87 | 75 (58 - 88) | 76 (59 - 95) | 78 (63 - 87) | 69 (51 - 82) | **0.057** |

**Supp. Table 2** Primary and secondary Endpoints, RRT separately

Endpoint data given as number of patients (percentage of patients). P-values were calculated using Chi-square-test. The p-value is reported in bold if the differences are statistically significant (p<0,05). Abbreviations: eGFR: estimated glomerular filtration rate, min.: minute, RRT: renal replacement therapy, d0: day 0, ICU: intensive care unit. All endpoints but ICU Survival include data for day 0-7 only

| Endpoint | Total | eGFR >60  ml/min/1.73m^2^ | eGFR 60-30  ml/min/1.73m^2^ | eGFR <30  ml/min/1.73m^2^ No RRT d0 | eGFR <30  ml/min/1.73m^2^ RRT d0 | p-value  column  4 vs. 5 |
| --- | --- | --- | --- | --- | --- | --- |
| Number of patients | 368 | 110 | 130 | 94 | 34 |  |
| Column | 1 | 2 | 3 | 4 | 5 |  |
| **Combined endpoint** | **239 (64.9%)** | **68 (61.8%)** | **82 (63.1%)** | **65 (69.1%)** | **24 (70.6%)** | **1** |
| Supraventricular tachycardias | 155 (42.1%) | 47 (42.7%) | 52 (40.0%) | 38 (40.4%) | 18 (52.9%) | 0.231 |
| Ventricular tachycardias | 71 (19.3%) | 19 (17.3%) | 29 (22.3%) | 19 (20.2%) | 4 (11.8%) | 0.312 |
| Death d0-7 | 90 (24.5%) | 17 (15.5%) | 31 (23.8%) | 31 (33.0%) | 11 (32.4%) | 1 |
| ICU Survival | 216 (58.7%) | 76 (69.1%) | 75 (57.7%) | 46 (48.9%) | 19 (55.9%) | 0.551 |

**Supp. Table 3** Characteristics of the matched cohort

Characteristics of patients with eGFR <30ml/min. and the propensity score matched cohort given as number of patients (percentage of patients) or as median (interquartile range). P-values were calculated using Fisher’s exact or Mann-Whitney test as applicable. The p-value is reported in bold if the differences are statistically significant (p<0,05). The caliper was set at 0.2. Abbreviations: eGFR: estimated glomerular filtration rate, min.: minute, ECLS: extracorporeal life support, PVAD: percutaneous ventricular assist device, RRT: renal replacement therapy

| Baseline characteristics | eGFR >30  ml/min/1.73m^2^ (N=113) | eGFR <30  ml/min/1.73m^2^ (N=113) | p-value |
| --- | --- | --- | --- |
| Male gender | 85 (75.2%) | 82 (72.6%) | 0.762 |
| Age [year] | 60 (53 - 72) | 66 (57 - 75) | **0.014** |
| Mechanical ventilation [h] | 151 (47 - 307) | 114.5 (0.75 - 279.75) | 0.071 |
| Coronary heart disease | 68 (60.2%) | 68 (60.2%) | 0.999 |
| Cardiomyopathy | 35 (31%) | 52 (46%) | **0.028** |
| Chronic heart failure | 54 (47.8%) | 76 (67.3%) | **0.005** |
| Chronic arrhythmias | 21 (18.6%) | 60 (53.1%) | **0.001** |
| Diabetes mellitus | 21 (18.6%) | 44 (38.9%) | **0.001** |
| Multimorbid | 15 (13.3%) | 46 (40.7%) | **0.001** |
| ECLS | 60 (53.1%) | 43 (38.1%) | **0.032** |
| PVAD | 24 (21.2%) | 9 (8%) | **0.008** |
| RRT | 33 (29.2%) | 52 (46%) | **0.013** |

**Suppl. Figure 1** Comparison of mean arterial pressure and norepinephrine rates

Mean arterial pressure (circle symbols, scaling on left y-axis) and norepinephrine rates (triangle symbols, scaling on right y-axis) shown as median and interquartile range.
